# Limited clinical value of two consecutive post-transplant renal scintigraphy procedures

**DOI:** 10.1007/s00330-019-06334-1

**Published:** 2019-07-23

**Authors:** Stan Benjamens, Robert A. Pol, Stefan P. Berger, Andor W. J. M. Glaudemans, Petra Dibbets-Schneider, Riemer H. J. A. Slart, Lioe-Fee de Geus-Oei

**Affiliations:** 1 Department of Radiology, Division of Nuclear Medicine, Leiden University Medical Center, Leiden University, Leiden, The Netherlands; 2 Department of Surgery, Division of Transplant Surgery, University Medical Center Groningen, University of Groningen, P.O. Box 30 001, 9700 RB Groningen, The Netherlands; 3 Medical Imaging Center, Department of Nuclear Medicine and Molecular Imaging, University Medical Center Groningen, University of Groningen, Groningen, The Netherlands; 4 Department of Internal Medicine, Division of Nephrology, University Medical Center Groningen, University of Groningen, Groningen, The Netherlands; 5 Department of Biomedical Photonic Imaging, MedTech Centre, University of Twente, Enschede, The Netherlands

**Keywords:** Radionuclide imaging, Kidney transplantation, Delayed graft function, Length of stay

## Abstract

**Objectives:**

Duration of delayed graft function (DGF) and length of hospital stay (LOS) are outcomes of interest in an era that warrants increased efficacy of transplant care whereas renal allografts originate increasingly from marginal donors. While earlier studies investigate the predictive capability of a single renal scintigraphy, this study focuses on the value for both DGF duration and LOS of consecutively performed scintigraphies.

**Methods:**

From 2011 to 2014, renal transplant recipients referred for a Tc-99m MAG3 renal scintigraphy were included in a single-center retrospective study. Primary endpoints were DGF duration and LOS. Both the first (≤ 3 days) and second scintigraphies (3–7 days after transplantation) were analyzed using a 4-grade qualitative scale and quantitative indices (*TFS*, *cTER*, *MUC10*, *average upslope*).

**Results:**

We evaluated 200 first and 108 (54%) consecutively performed scintigraphies. The Kaplan-Meier curves for DGF duration and qualitative grading of the first and second scintigraphy showed significant differences between the grades (*p* < 0.01). The Kaplan-Meier curve for the delta grades between these procedures (lower, equal, or higher grade) did not show significant differences (*p* = 0.18). Multivariate analysis showed a significant association between the qualitative grades, from the first and second scintigraphy, and DGF duration, HR 1.8 (1.4–2.2, *p* < 0.01) and 2.8 (1.8–4.3, *p* < 0.01), respectively.

**Conclusions:**

Qualitative grades of single renal scintigraphies, performed within 7 days after transplantation, can be used to make a reliable image-guided decision on the need for dialysis and to predict LOS. A consecutive renal scintigraphy, however, did not show an additional value in the assessment of DGF.

**Key Points:**

*• Post-transplant renal scintigraphy procedures provide information to predict delayed graft function duration and length of hospital stay*.

*• Performing two consecutive renal scintigraphy procedures within 1 week after transplantation does not strengthen the prediction of delayed graft function duration and length of hospital stay.*

*• Single renal scintigraphy procedures can be used to provide clinicians and patients with a reliable indication of the need for dialysis after transplantation and the expected duration of hospitalization.*

**Electronic supplementary material:**

The online version of this article (10.1007/s00330-019-06334-1) contains supplementary material, which is available to authorized users.

## Introduction

The duration of delayed graft function (DGF) and the length of hospital stay (LOS) are outcomes of interest in an era that warrants increased efficacy of transplant care whereas renal allografts originate increasingly from marginal donors, being allografts from extended criteria and donation after circulatory death (DCD) donors.

DGF describes the failure of the renal transplant to function immediately after transplantation [1]. DGF is associated with renal allograft failure in the first year after donation after brain death (DBD) transplantation; however, allografts with DGF still provide survival benefit compared to maintenance dialysis [2]. Moreover, DGF is associated with a higher incidence of biopsy-proven acute rejection and increased LOS [3]. The current trend of using marginal donors is associated with more DGF, longer hospital stay, and subsequently higher transplant-related costs [4–6]. Predicting the duration of DGF and LOS provides clinicians with the opportunity to optimize timing of renal biopsies and post-transplant dialysis. For this purpose, research focus has been on urinary and blood biomarkers for DGF, such as urinary tissue inhibitor of metalloproteinases-2 (TIMP-2), and quantitative/qualitative renal scintigraphy indices [7, 8].

Renal scintigraphy is an imaging biomarker of renal function, reflecting perfusion, reabsorption, and excretion. It may help predicting DGF and LOS [9, 10]. Results of renal scintigraphy can be interpreted qualitatively, differentiating in six- (*Heaf and Iversen grading scale*) or in four-curve types, and quantitatively, using several time-activity indices [11–16]. Several studies showed promising results for the use of renal scintigraphy to predict the course of DGF; however, these studies did not adjust for clinical variables associated with DGF [13, 17–20]. Moreover, previous studies focused primarily on the qualitative and quantitative interpretation of renal scintigraphy parameters from single procedures, whereas clinicians may focus more on consecutively performed imaging.

In this center, Technetium-99m mercaptoacetyltriglycine (Tc-99m MAG3) renal scintigraphies were performed consecutively in the first week after transplantation in all patients with ongoing DGF, according to a standard post-transplant protocol. The present study was initiated to determine if two consecutive renal scintigraphies improved the prediction of DGF and LOS.

## Materials and methods

### Study design and participants

We studied all patients receiving a renal transplant at the Leiden University Medical Center, between 2011 and 2014, who underwent a Tc-99m MAG3 renal scintigraphy within 3 days after transplantation. These patients are all part of a larger dual-center retrospective cohort, resulting in an earlier publication focusing on the predictive value of a single renal scintigraphy for the duration of DGF > 7 days after transplantation [21]. Patients were not included in case of receiving a dual renal transplant or both renal and pancreas transplants, and when under 18 years of age at the moment of transplantation. All clinical data for this study were retrieved from our national transplant research database, the Dutch Organ Transplant Registry (NOTR). Missing data and information on possible peri- and post-operative complications was retrieved by screening patients’ charts retrospectively. Patient data were processed and electronically stored according to the Declaration of Helsinki Ethical principles for medical research involving human subjects, and approval for this study was given by the Leiden University Medical Center ethics committee. The clinical and research activities being reported are consistent with the Principles of the Declaration of Istanbul as outlined in the “Declaration of Istanbul on Organ Trafficking and Transplant Tourism.”

### Outcome assessment

We defined DGF as the need for dialysis after transplantation (dialysis-based DGF) and as the failure of serum creatinine to decrease with ≥ 10%/day during 3 consecutive days (functional DGF), which is in accordance with the majority of studies on DGF [22]. Based on these definitions, we described early transplant function using four groups, namely *immediate graft function* (IGF), a serum creatinine decrease of ≥ 10%/day during 3 consecutive days or no need for dialysis; *slow graft function* (SGF), DGF between day 3–6 after transplantation; *delayed graft function* (DGF), DGF for more than 7 days after transplantation; *primary non-function* (PNF), immediate graft failure with the need of dialysis. We defined LOS as the number of days between transplantation and initial discharge.

### Clinical covariates

The following covariates were examined: (*i*) recipient factors (gender; age (years); body mass index (BMI, in kg/m^2^), diabetes mellitus, duration of pre-transplant dialysis (months)); (*ii*) donor factors (age (years), living (un)related (L(U)RD)) donation, DCD, DBD; (*iii*) transplant factors (pre-emptive transplantation, number of human leukocyte antigen (HLA) mismatches); (*iiii*) acute rejection, defined as renal biopsy-proven acute rejection (BPAR) or as non-BPAR, being an acute rejection treatment episode without BPAR according to Banff 2015 criteria [23].

### Renal scintigraphy

All included patients underwent renal scintigraphy for the analysis of DGF, discerning possible acute tubular necrosis from vascular or urological complications. In our center, a second renal scintigraphy was performed in case of ongoing DGF or suspicion of vascular/urological complications. Renal scintigraphies were performed using a bolus intravenous injection of 100 MBq Tc-99m MAG3. Two-phase digital dynamic images were obtained and processed using Syngo.via (Siemens Healthineers): (*i*) 1-s frames for 2 min; (*ii*) 20-s frames for 28 min. To calculate the renal scintigraphy time-activity curves, renal transplant regions-of-interest (ROIs) were drawn manually surrounding the renal transplant and the background ROIs were drawn crescent-shaped, opposite of the renal vessels. The analysis of the renal scintigraphy data was performed blinded to all clinical variables by a single researcher.

**Fig. 1:**
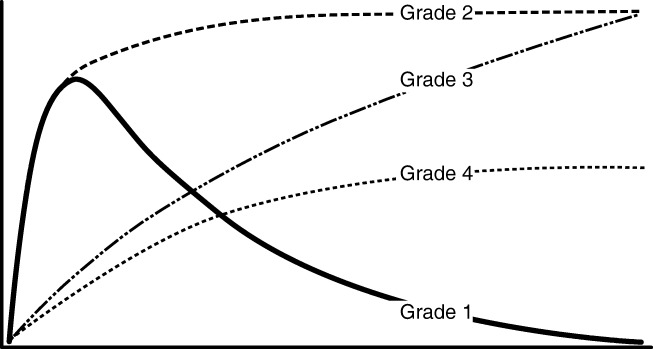
Qualitative renal scintigraphy grading: *grade 1*, a normal renal function with fast uptake and excretion; *grade 2*, a normal uptake with flat excretion curve; *grade 3*, a rising curve without excretion phase; *grade 4*, a reduced absolute uptake without excretion phase [21]

Qualitative analysis of the time-activity curves was performed using a four-curve type differentiation (Fig. 1 and Supplement Fig. 1A and B), with a normal renal function with fast uptake and excretion (*grade 1*), a normal uptake with flat excretion curve (*grade 2*), a rising curve without excretion phase (*grade 3*), and a reduced absolute uptake without excretion phase (*grade 4*). Furthermore, renal scintigraphy results were stratified into four groups, namely peri-transplant fluid collections, vascular complications, urological complications, and no complications.

Quantitative analysis was performed using four indices reflecting renal perfusion, reabsorption, and excretion. The *tubular function slope* (*TFS*) is a linear fit of the Tc-99m MAG3 curve between 50 and 110 s, reflecting the tracer uptake by renal tubular cells (counts/s) [13, 24]. *MUC10* reflects the uptake within the first 10 min, as a fraction of the injected dose (counts/s/MBq) [19]. The *corrected tubular extraction rate* (*cTER*) is the tracer uptake between procedure start and 2 min, corrected for the body surface (mL/min/1.73 m^2^) [17]. The *average upslope* reflecting the curve during the upslope period (counts at 3 min − counts at 20 s)/160 s, in counts/s) [21].

### Statistical analysis

Baseline descriptive statistics and clinical characteristics are presented as mean ± SD or median (range) for continuous variables and counts with percentages for categorical variables. The Mann-Whitney test and one-way ANOVA were used to describe the variance of continuous variables between groups. Two-sided *p* values of less than 0.05 were considered to indicate statistical significance. Correlations were assessed by means of Pearson’s or Spearman’s analysis. Univariate and multivariate Cox proportional hazards analysis and the Kaplan-Meier curves with log-rank tests were used to examine the associations. The hazard ratios (HRs) and their corresponding 95% confidence intervals (CIs) are reported. The added value of renal scintigraphy indices was assessed by examining the change in − 2 log likelihood. We used Package for the Social Sciences (IBM^©^ SPSS Statistics^©^ version 22) for all statistical analyses and GraphPad Prism, version 5.04 (GraphPad Software), for graph presentation.

## Results

### Patient characteristics

Patients’ characteristics of the 200 included patients are displayed in Table 1. Median age was 52 ± 13 years, 59% were male, 12% underwent pre-emptive renal transplantation, and median (IQR) duration of pre-transplant dialysis was 36.4 (14.3–57.3) months. Seventy-four (37%) patients received a DBD transplant, 94 (47%) a DCD transplant, and 32 (16%) a living donor transplant. The median (IQR) LOS was 15 (11–21) days.

**Table 1:** Patient characteristics

Variable	Patients (*n* = 200)
Male	116 (59)
Age, years^b^	55 ± 13
BMI^b^	26.6 ± 3.7
Pre-emptive Txa	24 (12)
Duration pre-Tx dialysis, months ^c^	36.4 (14.3–57.3)
Type of donation	
Living (un)related	32 (16)
DBD	74 (37)
DCD	94 (47)
DGF > 7 days after Tx^a^	131 (66)
Length of hospital stay (days)^c^	15 (11–21)
Rejection^a^	
7 days after Tx	22 (11)
14 years after Tx	37 (18.5)

*Tx* kidney transplantation, *DGF* delayed graft function, *DBD* donation after brain death, *DCD* donation after circulatory death

^a^*n* (%)

^b^Mean ± standard deviation (SD)

^c^Median (IQR)

For 161 (81%) patients, the indication for the first renal scintigraphy was a suspected acute tubular necrosis as cause of DGF. For 39 (19%) patients, the indication was a suspicion for fluid collections and vascular or urological complications. Only 3 out of these 39 patients experienced a vascular or urological complication needing a surgical intervention within 2 weeks after transplantation.

**Fig. 2:**
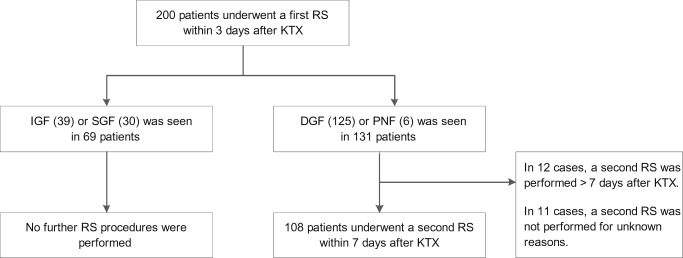
Flowchart of the included patients

The study population was stratified into four groups, based on early transplant function, as shown in Table 2. From the 131 patients experiencing either DGF or PNF, 108 patients underwent a second renal scintigraphy within 7 days after transplantation (Fig. 2).

**Table 2:** Early graft function for different types of kidney donation

	Early graft function			
	IGF (*n* = 39)	SGF (*n* = 30)	DGF (125)	PNF (*n* = 6)
Type of donation				
Living (un)related	20 (63%)	2 (6%)	7 (22%)	3 (9%)
DBD	16 (22%)	16 (22%)	41 (55%)	1 (1%)
DCD	3 (3%)	12 (13%)	77 (82%)	2 (2%)

*IGF* immediate graft function, *SGF* slow graft function, *DGF* delayed graft function, *PNF* primary non-function, *DBD* donation after brain death, *DCD* donation after circulatory death

### Qualitative grades and DGF duration

The qualitative grades of the first renal scintigraphy did significantly (*p* < 0.01) differ between the groups of early graft dysfunction. DGF was observed in 75 (81%) out of 93 patients with grade 3 and in 35 (85%) out 41 patients with grade 4, while IGF was noticed in 16 (88%) out of 18 patients with grade 1 and in 19 (40%) out of 48 patients with grade 2 (Supplement Table 1).

**Fig. 3:**
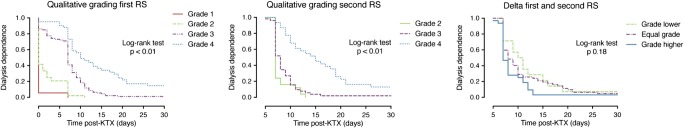
The Kaplan-Meier curves for qualitative grading

The Kaplan-Meier curves (Fig. 3) for the duration of DGF and qualitative grading of the first and second renal scintigraphy showed a significant difference in DGF duration between grade 2, grade 3, and grade 4 (*p* < 0.01) and between grade 3 and grade 4 (*p* < 0.01), respectively. The Kaplan-Meier curve for delta qualitative grades between the first and second renal scintigraphies did not show significant differences between grades (*p* = 0.18).

Using the univariate Cox proportional hazards analysis, qualitative grades of both the first and second renal scintigraphies were significantly associated with the DGF duration. The delta qualitative grades between the first and second renal scintigraphies were not significantly associated with the duration of DGF (Table 4).

Based on the qualitative grades, the anticipated moment of DGF ending was calculated in a subset of patients without IGF (Table 3): *grades 1* and *2* of the first renal scintigraphy correspond with a median (IQR) of 5.0 (2.0–7.0) days DGF; *grade 3* with 7.0 (6.3–10.0) days DGF; *grade 4* with 11.0 (7.5–19.5) days DGF. Outcomes corresponding with the qualitative grading of the second renal scintigraphy are presented in Table 3.

**Table 3:** Duration of delayed graft function and length of hospital stay based on qualitative grading

		Duration of DGF		Length of hospital stay	
	Patients, *n*	Median	IQR	Median	IQR
First					
Grades 1 and 2	30	5.0	2.0–7.0	11.0	12.0–19.0
Grade 3	89	7.0	6.3–10.0	15.0	12.0–19.0
Grade 4	41	11.0	7.5–19.5	20.0	14.0–28.5
Second					
Grade 2	25	7.0	7.0–7.5	14.0	12.0–16.5
Grade 3	52	8.0	7.0–10.0	15.0	12.3–19.0
Grade 4	31	15.0	10.0–20.0	22.0	15.8–29.3

Results based on a subset of the included patients, after exclusion of all patients with immediate graft function

### Quantitative indices and DGF duration

Quantitative indices *TFS*, *cTER*, and *average upslope* of the first renal scintigraphy were significantly different between IGF and SGF, whereas *MUC10* did not show a significant difference. All indices were significantly different between SGF and DGF, whereas no significant difference was observed between DGF and PNF (Supplement Table 1 and Supplement Fig. 2).

For the first renal scintigraphy, there was a significant association between the quantitative indices and DGF duration: *TFS*, *r* = − 0.44, *p* < 0.01; *MUC10*, − 0.46, *p* < 0.01; *cTER*, − 0.44, *p* < 0.01; *average upslope*, − 0.45, *p* < 0.01. The analysis of the second renal scintigraphy showed a weaker, but still significant association between the quantitative indices and DGF duration: *TFS*, *r* = − 0.32, *p* = 0.01; *MUC10*, − 0.30, *p* = 0.02; *cTER*, − 0.32, *p* = 0.01; *average upslope*, − 0.33, *p* = 0.01. The analysis of the delta quantitative indices between the first and second renal scintigraphies showed an even weaker association: *TFS*, *r* = − 0.24, *p* = 0.01; *MUC10*, − 0.26, *p* < 0.01; *cTER*, − 0.24, *p* = 0.01; *average upslope*, − 0.25, *p* = 0.01.

Using the univariate Cox proportional hazards analysis, the quantitative indices of both the first and second renal scintigraphies were significantly associated with the duration of DGF. The deltas of the quantitative indices *TFS* and *cTER*, between the first and second renal scintigraphies, were significantly associated with the duration of DGF, HR 0.4 (0.4–0.8, *p* < 0.01) and HR 1.0 (1.0–1.0, *p* < 0.01), respectively (Table 4).

**Table 4:** Cox proportional hazards analysis for quantitative/qualitative indices and duration of delayed graft function

	First renal scintigraphy (*n* = 200)				Second renal scintigraphy (*n* = 108)				Delta renal scintigraphy (*n* = 108)			
Indices	Univariate		Multivariate		Univariate		Multivariate		Univariate		Multivariate	
	Hazard ratio	*p* value	Hazard ratio	*p* value	Hazard ratio	*p* value	Hazard ratio	*p* value	Hazard ratio	*p* value	Hazard ratio	*p* value
Quantitative												
TFS	0.5 (0.4–0.6)	< 0.01	1.3 (0.3–6.0)	0.77	0.6 (0.4–0.8)	< 0.01	2.3 (0.4–12.4)	0.34	0.4 (0.2–0.8)	< 0.01	0.4 (0.1–1.4)	0.16
MUC10	0.2 (0.1–0.4)	< 0.01	0.5 (0.2–1.2)	0.12	0.4 (0.2–0.8)	< 0.01	1.0 (0.2–3.9)	0.97	1.0 (1.0–1.0)	0.09	1.0 (1.0–1.0)	0.88
cTER	0.6 (0.5–0.7)	< 0.01	0.8 (0.2–2.6)	0.68	0.6 (0.4–0.8)	< 0.01	0.3 (0.1–2.1)	0.45	1.0 (1.0–1.0)	< 0.01	1.0 (1.0–1.0)	0.16
Average upslope	0.6 (0.5–0.8)	< 0.01	0.9 (0.7–1.2)	0.59	0.5 (0.3–0.8)	< 0.01	0.9 (0.4–2.1)	0.73	1.0 (1.0–1.0)	0.83	1.0 (1.0–1.0)	0.77
Qualitative grading (0–4)	2.0 (1.7–2.4)	< 0.01	1.8 (1.4–2.2)	< 0.01	2.2 (1.6–3.0)	< 0.01	2.8 (1.8–4.3)	< 0.01	1.3 (1.0–1.8)	0.08	1.4 (0.9–2.2)	0.15

Data in parentheses are 95% confidence intervals. Hazard ratios are per log-unit of change for the quantitative indices. Multivariate analysis consists of all quantitative/qualitative indices, recipient age, gender, body mass index, diabetes mellitus, DCD donation, pre-emptive transplantation, donor age, HLA mismatches, and duration of pre-transplant dialysis

*TFS* tubular function slope, *MUC10* first 10-min uptake as a fraction of the injected dose, *cTER* corrected tubular extraction rate, *Average upslope* the slope during counts at 20 s and counts at 3 min

### Qualitative grades and length of hospital stay

Using the univariate Cox proportional hazards analysis, qualitative grades of the first renal scintigraphy were significantly associated with LOS (Supplement Table 3). Based on the qualitative grades, the anticipated LOS was calculated (Table 3): *grades 1* and *2* of the first renal scintigraphy correspond with a median (IQR) of 11.0 (12.0–19.0) days of hospitalization; *grade 3* with 15.0 (12.0–19.0) days of hospitalization; *grade 4* with 20.0 (14.0–28.5) days of hospitalization. Outcomes corresponding with the qualitative grading of the second renal scintigraphy and the delta between the first and second renal scintigraphies are presented in Table 3.

### Quantitative indices and length of hospital stay

For the first renal scintigraphy, there was a significant, however, weak correlation between LOS and the quantitative indices: *TFS*, *r* = − 0.23, *p* < 0.01; *MUC10*, − 0.28, *p* < 0.01; *cTER*, − 0.23, *p* < 0.01; *average upslope*, − 0.19, *p* < 0.01. The analysis of the second renal scintigraphy also showed a weak but significant correlation between LOS and the quantitative indices: *TFS*, *r* = − 0.24, *p* = 0.02; *MUC10*, − 0.26, *p* < 0.01; *cTER*, − 0.24, *p* = 0.02; *average upslope*, − 0.23, *p* = 0.02. Using the univariate Cox proportional hazards analysis, the quantitative indices (*TFS*, *MUC10*, *and cTER*) of the first renal scintigraphy were significantly associated with LOS (Supplement Table 3).

### Multivariate analysis

Covariates with a significant association with DGF were DCD donation, HR 1.9 (1.4–2.5, *p* < 0.01); pre-emptive transplantation, HR 0.5 (0.3–0.7, *p* < 0.01); and duration of pre-transplant dialysis, HR 1.1 (1.0–1.1, *p* < 0.01) (Supplement Table 3). The clinical covariates recipient gender, recipient age, recipient BMI, pre-transplantation diabetes mellitus, donor age, and HLA mismatches did not contribute to a significant hazard ratio (Supplement Table 2). Outcomes corresponding with the LOS are presented in Supplement Table 2.

In a multivariate analysis, including all other quantitative indices, the qualitative grading scale, and the clinical covariates, the association between the qualitative grading of the first renal scintigraphy and the duration of DGF was significant for grade 3, HR 2.3 (1.3–4.2, *p* < 0.01), and grade 4, HR 3.4 (1.7–7.1, *p* < 0.01). The association between the qualitative grading of the second renal scintigraphy and the duration of DGF was significant for grade 4, HR 4.1 (1.9–8.8, *p* < 0.01) (Table 4).

In a multivariate analysis, including all other quantitative indices, the qualitative grading scale, and the clinical covariates, the association between the qualitative grading of the first renal scintigraphy and LOS was HR 1.3 (1.0–1.6, *p* = 0.04). Multivariate analysis of the quantitative indices, the qualitative grading scale of the second renal scintigraphy, and LOS did not result in significant associations (Supplement Table 2).

### Predictive performance of qualitative grades for the duration of DGF

When assessing the predictive performance of the clinical variables, the − 2 log likelihood improved significantly when including the qualitative grades from the first renal scintigraphy (1623.6 to 1583.7, *p* < 0.01). The predictive performance of the model with clinical variables did not show a significant improvement after including the qualitative grades of the second renal scintigraphy (766.0 to 737.9, *p* < 0.01).

## Discussion

Our analysis of Tc-99m MAG3 renal scintigraphy indicates that qualitative grades of two separately analyzed procedures, at ≤ 3 and ≤ 7 days after transplantation respectively, are significantly associated with DGF duration and the LOS. However, the delta of qualitative grades and the changes of quantitative indices between these sequential performed renal scintigraphies are not associated with the duration of DGF and the LOS. These findings underline the strength of the qualitative analysis of a single renal scintigraphy for the prediction of DGF duration and LOS. Conversely, there is no additional value of performing repetitive renal scintigraphy procedures to assess DGF and LOS.

Our study confirms the findings of previous studies, which indicated the applicability of the quantitative indices *TFS*, *MUC10*, *cTER* and *average upslope* and of the qualitative grading with four or six grades for the evaluation of DGF [12, 13, 17–20]. A previous study, focusing on *TFS* at 48 h after transplantation showed the capability of this index to separate patients with DGF from patients with IGF [13]. In our study, *TFS* differed significantly between types of early transplant function and was associated with a longer duration of DGF, HR 0.5 (0.4–0.6, *p* < 0.01) and HR 0.6 (0.4–0.8, *p* < 0.01) respectively for the first and second renal scintigraphy. For *MUC10* from a renal scintigraphy performed within 48 h after transplantation, a previous study showed significant differences between DGF and non-DGF patients, which is in line with the results of our analysis, showing a significant difference in *MUC10* values between the SGF and DGF groups [19]. For *cTER* from a renal scintigraphy performed ≤ 4 days after transplantation, a previous study showed a significant correlation with the period of dialysis dependence (*r* = − 0.68, *p* < 0.01), which is slightly stronger than the correlation found in this study (*r* = − 0.44, *p* < 0.01) [17]. In a previous study, a four-grade index was introduced for renal scintigraphy at ≤ 3 days after transplantation, using this four-grade index, an independent association between a longer duration of DGF and the qualitative grades was shown, HR 1.8 (1.4–2.2, *p* < 0.01), which is consistent with the results of studies using both four- and six-grade indices [12, 21].

Although previous studies have described the applicability of a first renal scintigraphy at ≤ 48 or 62 h after transplantation, this study is the first comprehensive analysis of a second renal scintigraphy at ≤ 7 days after transplantation. Quantitative indices of the second renal scintigraphy were associated with the duration of DGF, however, not when adjusted for clinical covariates. Multivariate analysis of the first and second renal scintigraphy showed an independent significant association between the qualitative grades and duration of DGF, HR 1.8 (1.4–2.2, *p* < 0.01) and HR 2.8 (1.8–4.3, *p* < 0.01), respectively. The delta qualitative grades between the procedures were not significantly associated with the duration of DGF in multivariate analysis.

The presented results should be evaluated in light of non-imaging biomarkers for DGF, such as the urinary biomarker TIMP-2 and neutrophil gelatinase-associated lipocalin (NGAL). The predictive value of TIMP-2 was assessed in a population of DCD transplant recipients (*n* = 74), showing an area under the curve (AUC) of 0.89 (95% CI 0.78–0.99) for > 7 days functional DGF [7]. For urinary NGAL, the AUC for > 7 functional and dialysis-based DGF was 0.75 (95% CI 0.65–0.84) in a population of both DBD and DCD transplant recipients (*n* = 176) [8]. Renal scintigraphy, performed within 3 days post-transplantation to predict ≥ 7 functional and dialysis-based DGF, showed to have an 87% sensitivity and 65% specificity when analyzed qualitatively and an AUC of 0.82 (95% CI 0.78–0.86) when analyzed quantitatively [21]. Further prospective studies are needed to establish the clinical value of qualitative and quantitative renal scintigraphy analysis in light of emerging non-imaging biomarkers for DGF.

Previous studies focusing on the use of renal scintigraphy after transplantation did not use LOS as one of the endpoints. However, this is important since with the increased use of renal allografts from extended criteria and DCD donors, a prolonged hospital stay, and subsequent higher transplant-related costs are reported [4]. In addition, an increased focus on patient-related outcome measures (PROMS) shows the significance of informing patients on the clinical path during and after hospitalization, urging for a reliable indication of moment of DGF ending and the LOS. The results of our study are in line with the literature, with 47% of the transplants coming from DCD donors, a median length of stay of 15 [11–21] days, and in 66% of patients a DGF duration of > 7 days, and provide insight in the expected moment of hospital discharge.

Due to the retrospective design, a clinical selection bias resulted in a cohort of patients with a high incidence of DGF and a minimal number of patients with IGF, this selection further increased when analyzing patients with a second renal scintigraphy. On the contrary, the relatively large number of patients with a sequential renal scintigraphy ≤ 7 days after transplantation contributes to a reliable analysis. Thereby, we performed an extensive multivariate analysis to adjust for possible confounders, including all qualitative and quantitative scintigraphy indices in a single model. Presenting a single-center study with a small time frame for inclusion, we can expect a uniformity in transplant care and similarity in renal scintigraphy. Furthermore, analyzing the results quantitatively and qualitatively decreases the impact of inter-observer variability, while our blinded renal scintigraphy analysis decreases the risk of bias.

## Conclusion

In conclusion, a reliable indication of the duration of DGF and the LOS can be provided by qualitative analysis of single renal scintigraphy, whereas the qualitative and quantitative change between sequentially performed renal scintigraphies does not strengthen the prediction of DGF duration and LOS. Qualitative grades of single renal scintigraphy can be used to provide clinicians and patients with a reliable indication of the need for dialysis after transplantation and the expected duration of hospitalization, while the additional value of performing a consecutive renal scintigraphy for the assessment of DGF was not found.

## Electronic supplementary material

ESM 1 (DOCX 145 kb)

ESM 2 (PNG 58 kb)

High Resolution Image (EPS 719 kb)

ESM 3 (PNG 97 kb)

High Resolution Image (EPS 904 kb)

ESM 4 (PNG 128 kb)

High Resolution Image (EPS 87 kb)

## Abbreviations

DBD: Donation after brain death
DCD: Donation after circulatory death
DGF: Delayed graft function
IGF: Immediate graft function
LOS: Length of hospital stay
PNF: Primary non-function
SGF: Slow graft function
