# Supplementary material for: Limited clinical value of two consecutive post-transplant renal scintigraphy procedures
Source: Eur Radiol. 2019 Jul 23;30(1):452–60. doi: 10.1007/s00330-019-06334-1 (PMC6890596; doi:10.1007/s00330-019-06334-1)
Supplement: Supplementary file 1 — (DOCX 145 kb) [file 330_2019_6334_MOESM1_ESM.docx]

**Supplement Table 1: Distribution of quantitative and qualitative indices from the first renal scintigraphy between groups of early graft function**

|  | | **Early graft function** | | | | |
| --- | --- | --- | --- | --- | --- | --- |
| **Variable** | | **IGF**  (n = 39) | **SGF**  (n = 30) | **DGF**  (125) | **PNF**  (n = 6) | **p-value** |
| **Quantitative indices** ^a^ | |  |  |  |  |  |
|  | TFS | 1.00 (0.57 – 1.76) | 0.45 (0.29 – 0.68) | 0.26 (0.12 – 0.42) | 0.17 (0.02 – 0.74) | < 0.01 ^c^ |
|  | cTER | 601 (345 -1048) | 277 (183 – 419) | 164 (82 – 257) | 110 (21 – 448) | < 0.01 ^c^ |
|  | MUC10 | 1314 (956 – 2275) | 994 (665 – 1665) | 607 (393 – 967) | 306 (197 – 1395) | < 0.01 ^c^ |
|  | Average Upslope | 0.98 (0.57 – 1.60) | 0.53 (0.33 – 0.89) | 0.30 (0.14 – 0.55) | 1.13 (0.10 – 2.05) | 0.80 ^c^ |
| **Qualitative grading** ^b^ | |  |  |  |  |  |
|  | Grade 1 | 16 (88) | 1 (6) | 1 (6) | 0 (0) | < 0.01 ^d^ |
|  | Grade 2 | 19 (40) | 14 (29) | 14 (29) | 1 (2) | < 0.01 ^d^ |
|  | Grade 3 | 4 (4) | 14 (15) | 75 (81) | 0 (0) | < 0.01 ^d^ |
|  | Grade 4 | 0 (0) | 1 (2) | 35 (85) | 5 (12) | < 0.01 ^d^ |
| IGF = immediate graft function, SGF = slow graft function, DGF = delayed graft function, PNF = primary non-function  ^a^ median (IQR), ^b^ n (%), ^c^ P-value based on one-way ANOVA, ^d^ P-value based on chi-square test | | | | | |  |

**Supplement Table 2: Univariate Cox proportional hazard analysis of clinical covariates**

| **Indices** | | | **Duration of DGF** | | **Length of hospital stay** | |
| --- | --- | --- | --- | --- | --- | --- |
|  | | | Hazard ratio | p-value | Hazard ratio | p-value |
| **Recipients** | | |  |  |  |  |
|  | | Gender, male | 1.0 (0.7-1.3) | 0.84 | 1.0 (0.7-1.3) | 0.75 |
|  | | Age, years | 1.0 (1.0-1.0) | 0.10 | 1.0 (1.0-1.0) | 0.89 |
|  | | Duration pre-Tx dialysis, months | 1.1 (1.0-1.1) | **< 0.01** | 1.0 (1.0-1.0) | 0.55 |
|  | | BMI | 1.0 (1.0-1.0) | 0.76 | 1.0 (1.0-1.0) | 0.95 |
|  | | Diabetes mellitus | 1.0 (0.8-1.4) | 0.82 | 1.6 (1.1-2.2) | **< 0.01** |
|  | | Pre-emptive Tx | 0.5 (0.3-0.7) | **< 0.01** | 0.7 (0.5-1.2) | 0.20 |
| **Donor** | | |  |  |  |  |
|  | Age, years | | 1.0 (1.0-1.0) | 0.29 | 1.0 (1.0-1.0) | 0.58 |
|  | DCD | | 1.9 (1.4-2.5) | **< 0.01** | 1.1 (0.8-1.4) | 0.58 |
|  | HLA-mismatches | | 0.9 (0.9-1.1) | 0.33 | 1.1 (1.0-1.3) | **0.03** |

Tx = transplantation; BMI = Body Mass Index; DCD = donation after circulatory death; HLA-mismatches = human leukocyte antigen mismatches

**Supplement Table 3: Cox proportional hazard analysis for quantitative / qualitative indices and length of hospital stay**

|  | | **First renal scintigraphy**  (n = 200) | | | | | **Second renal scintigraphy**  (n = 108) | | | | **Delta renal scintigraphy**  (n = 108) | | | |
| --- | --- | --- | --- | --- | --- | --- | --- | --- | --- | --- | --- | --- | --- | --- |
| **Indices** | | | Univariate | | Multivariate | | Univariate | | Multivariate | | Univariate | | Multivariate | |
|  | | | Hazard ratio | p-value | Hazard ratio | p-value | Hazard ratio | p-value | Hazard ratio | p-value | Hazard ratio | p-value | Hazard ratio | p-value |
| **Quantitative** | | |  |  |  |  |  |  |  |  |  |  |  |  |
|  | **TFS** | | 0.6 (0.4-0.8) | **< 0.01** | 1.0 (1.0-1.0) | 0.14 | 0.7 (0.4-1.2) | 0.20 | 1.7 (0.4-6.9) | 0.57 | 1.2 (0.8-1.8) | 0.48 | 0.9(0.5-1.7) | 0.72 |
|  | **MUC10** | | 1.0 (1.0-1.0) | **< 0.01** | 1.0 (1.0-1.0) | 0.06 | 1.0 (1.0-1.0) | 0.14 | 1.0 (1.0-1.0) | 0.46 | 1.1(0.7-1.7) | 0.76 | 0.9(0.5-1.7) | 0.64 |
|  | **cTER** | | 1.0 (1.0-1.0) | **0.02** | 1.0 (0.9-1.0) | 0.30 | 1.0 (1.0-1.0) | 0.20 | 1.0 (1.0-1.0) | 0.14 | 1.3(0.8-1.9) | 0.37 | 0.9(0.5-1.7) | 0.72 |
|  | **Average Upslope** | | 1.0 (1.0-1.0) | 0.75 | 1.0 (1.0-1.0) | 0.25 | 0.7 (0.5-1.1) | 0.12 | 0.9 (0.3-3.7) | 0.82 | 1.1(0.7-1.7) | 0.61 | 1.0(0.6-1.8) | 0.87 |
| **Qualitative grading (0 – 4)** | | | 1.4 (1.2-1.6) | **< 0.01** | 1.3 (1.0-1.6) | **0.04** | 0.4 (1.1-1.9) | **0.01** | 1.4 (0.9-2.2) | 0.09 | 1.1(0.8-1.5) | 0.54 | 1.2(0.7-1.9) | 0.56 |
| Data in parentheses are 95% confidence intervals. Hazard ratios are per Log-unit of change for the quantitative indices.  Multivariate analysis consists of all quantitative/qualitative indices, recipients age, gender, Body Mass Index, Diabetes Mellitus, DCD donation, pre-emptive transplantation, donor age, HLA-mismatches, and duration of pre-transplant dialysis.  TFS = Tubular function slope, MUC10 = first 10 min uptake as a fraction of the injected dose, cTER = corrected tubular extraction rate,  Average upslope = reflecting the slope during counts at 20 sec and counts at 3 min. | | | | | | | | | | |  | |  | |

**
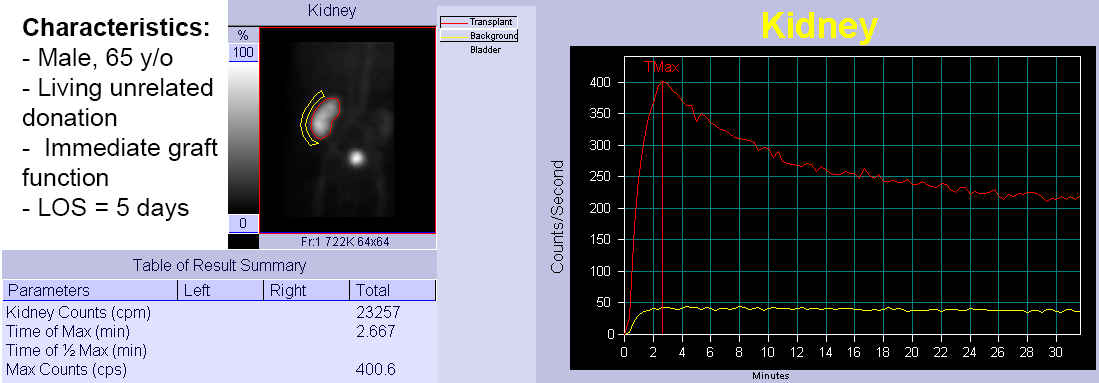
**

**Supplement Figure 1A:** Example post-transplant renal scintigraphy result, showing a *Grade 1* time-activity curve from a 65-year-old male transplant recipient. This living unrelated donation transplantation, resulted in immediate graft function, with a total length of hospital stay of 5 days.


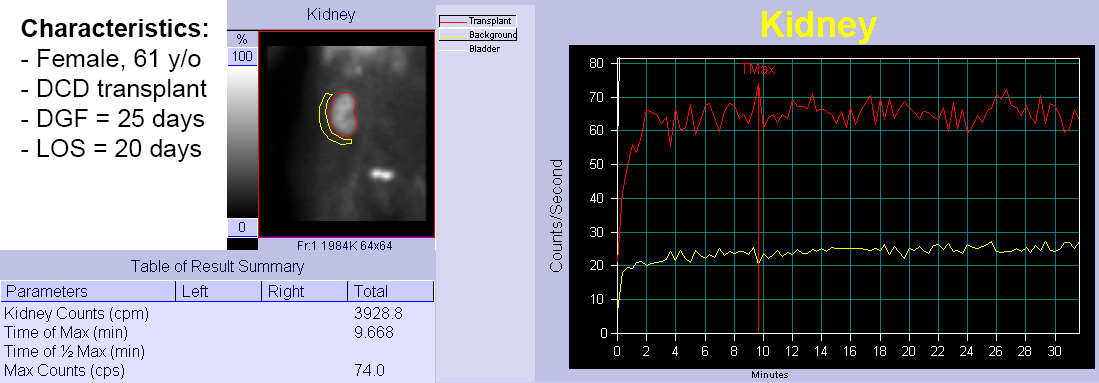


**Supplement Figure 1B:** Example post-transplant renal scintigraphy result, showing a *Grade 4* time-activity curve from a 61-year-old female transplant recipient. This donation after circulatory death transplantation, resulted in a delayed graft function of 25 days, with a total length of hospital stay of 20 days.

**
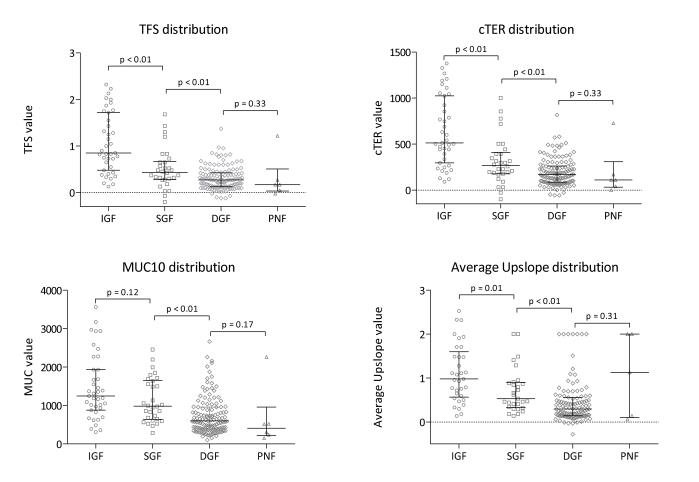
**

**Supplement Figure 2:** Distribution of quantitative and qualitative indices between groups of early graft function
