# Supplementary figures and images for: Limited clinical value of two consecutive post-transplant renal scintigraphy procedures
Source: Eur Radiol. 2019 Jul 23;30(1):452–60. doi: 10.1007/s00330-019-06334-1 (PMC6890596; doi:10.1007/s00330-019-06334-1)

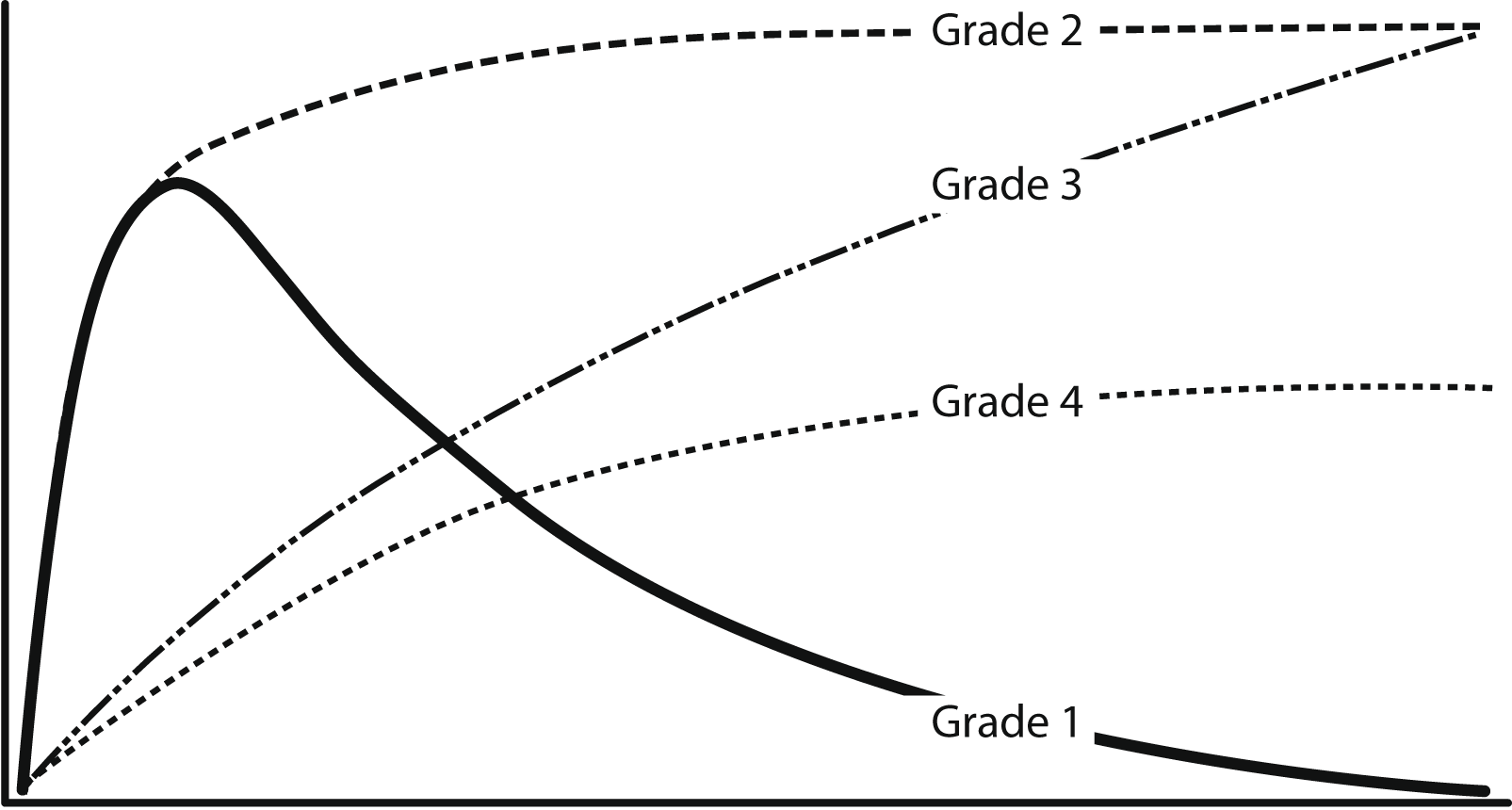

Supplement: Supplementary file 2 — (PNG 58 kb) [file 330_2019_6334_Fig4_ESM.png]

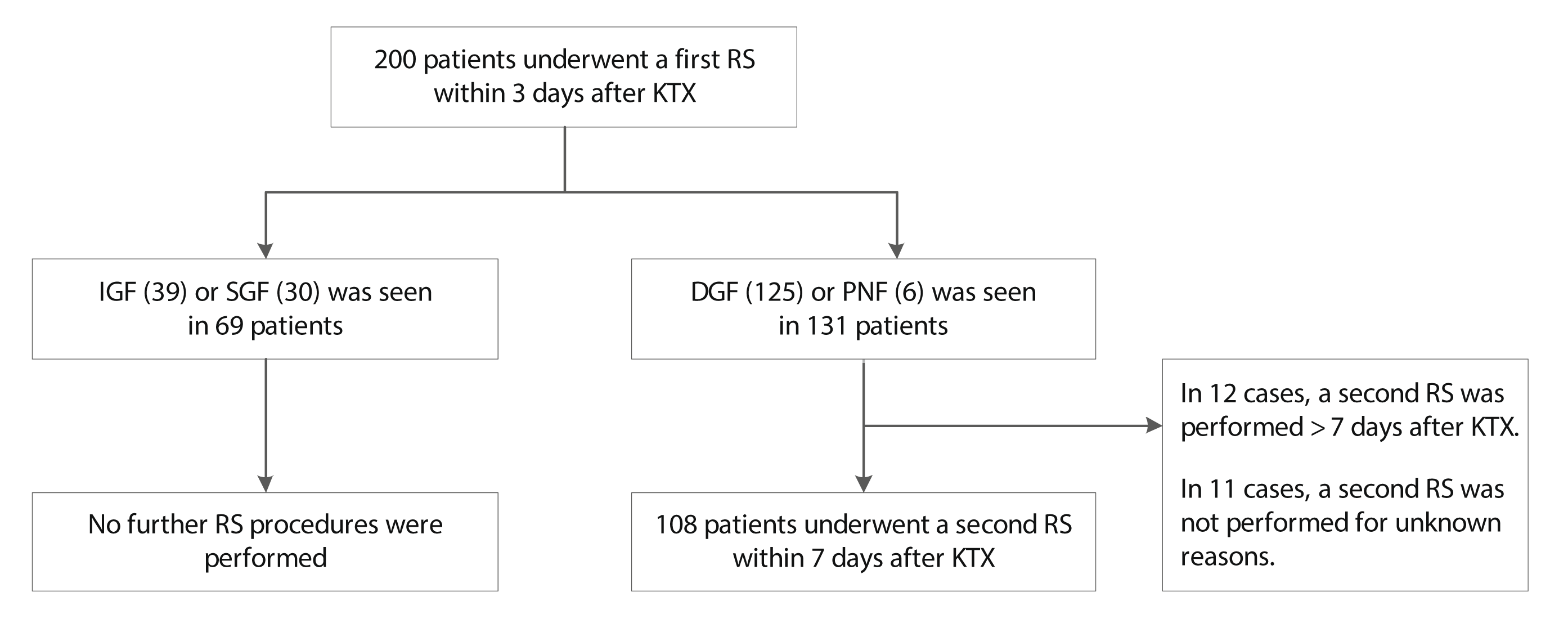

Supplement: Supplementary file 4 — (PNG 97 kb) [file 330_2019_6334_Fig5_ESM.png]

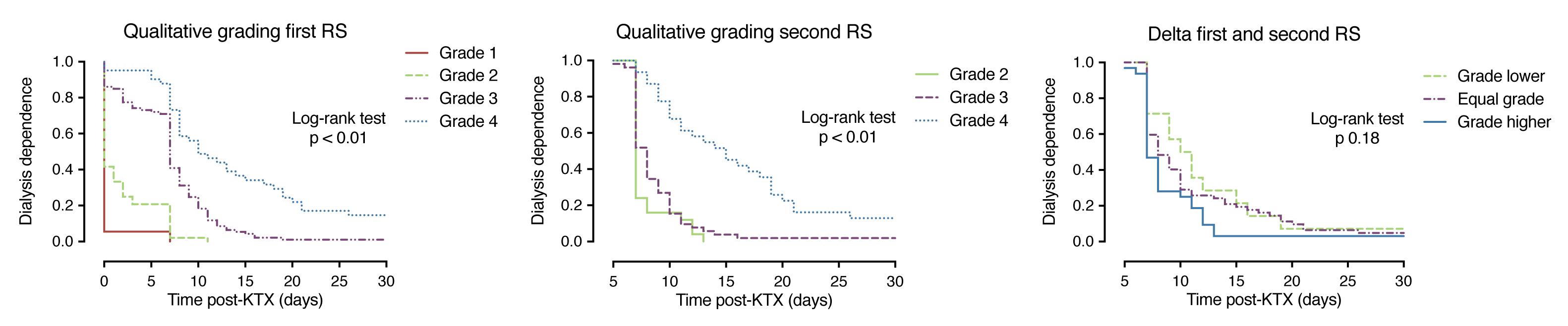

Supplement: Supplementary file 6 — (PNG 128 kb) [file 330_2019_6334_Fig6_ESM.png]
